# Long-term persistence and boostability of immune responses following different rabies pre-exposure prophylaxis priming schedules of a purified chick embryo cell rabies vaccine administered alone or concomitantly with a Japanese encephalitis vaccine

**DOI:** 10.1371/journal.pntd.0013118

**Published:** 2025-05-27

**Authors:** Tomas Jelinek, Mirjam Schunk, Emil C. Reisinger, Marylyn M. Addo, Ursula Wiedermann, Marco Costantini, Maria Lattanzi, Michele Pellegrini, Ilaria Galgani, on behalf of the Rabies-018 V49_23E1 study group

**Affiliations:** 1 Berlin Center for Travel and Tropical Medicine, Berlin, Germany; 2 Institute of Infectious Diseases and Tropical Medicine, LMU Klinikum, Munich, Germany; 3 Department of Tropical Medicine and Infectious Diseases, Rostock University Medical Center, Rostock, Germany; 4 Bernhard Nocht Centre for Clinical Trials (BNCCT), Bernhard Nocht Institute for Tropical Medicine, Centre for Internal Medicine, University Medical Center Hamburg-Eppendorf (UKE), Hamburg, Germany; 5 Institute of Specific Prophylaxis and Tropical Medicine, Center for Pathophysiology, Infectiology and Immunology, Medical University of Vienna, Vienna, Austria; 6 GSK, Siena, Italy; Public Health Agency of Canada, CANADA

## Abstract

**Background:**

Rabies pre-exposure prophylaxis (PrEP) is recommended to individuals at risk for exposure to rabies. Three intramuscular doses of the purified chick embryo cell (PCEC) rabies vaccine can be administered according to a conventional (four-week) or an accelerated (one-week) regimen.

**Methodology/Principal findings:**

This phase III, open-label study (NCT02545517) was an extension of the NCT01662440 study where immune responses of different primary PrEP regimens with PCEC rabies vaccine and Japanese encephalitis (JE) vaccine were assessed. Adults who had completed the parent study and received three doses of rabies PrEP regimens, concomitantly with a JE vaccine or alone (i.e., Rabies+JE-Accelerated, Rabies+JE-Conventional, and Rabies-Conventional groups) were enrolled in this extension study. Here we evaluated the long-term (up to 10 years after completing the primary vaccination) immunogenicity and boostability of PCEC rabies vaccine, and the safety of booster dose(s). Immunogenicity was assessed in terms of rabies virus neutralizing antibody (RVNA) concentrations, and titers ≥0.5 international units (IU)/mL were considered adequate for protection. Participants with RVNA concentrations <0.5 IU/mL were eligible for receiving PCEC rabies vaccine booster(s). Of the 459 participants enrolled in this study, 77.6% completed the trial. At the study end, the probability of detecting adequate RVNA concentrations in unboosted participants was 57.8%, 60.2%, and 62.0% for the Rabies+JE-Accelerated, Rabies+JE-Conventional, and Rabies-Conventional groups, respectively. Overall, 68.6% of all participants had RVNA concentrations ≥0.5 IU/mL at any timepoint and did not require a booster dose during the study follow-up period. Of the 144 participants with RVNA concentrations <0.5 IU/mL at any timepoint, 132 needed one booster dose throughout the follow-up period (Years 3–10) and 12 needed multiple booster administrations. No safety concerns were identified.

**Conclusion/Significance:**

The PCEC rabies vaccine administered alone/concomitantly with the JE vaccine provides adequate immunity for up to 62% of unboosted participants at study end.

## Author summary

Pre-exposure prophylaxis (PrEP) with the purified chick embryo cell (PCEC) rabies vaccine is recommended to people at risk. We conducted a phase III, open-label study (NCT02545517) as an extension of the NCT01662440 study, where we assessed immune responses elicited by different primary PrEP regimens (accelerated [one-week] or conventional [four-week]) with PCEC rabies vaccine administered alone or concomitantly with the Japanese encephalitis vaccine. In the extension study, we evaluated the long-term immunogenicity and boostability of the PCEC rabies vaccine after PrEP administration in the parent study, over a period of 3–10 years from primary vaccination. At study end, 58–62% of unboosted participants had an adequate response to vaccination based on antibody levels. During the follow-up period, 68.6% of all participants did not require a booster dose and most low/non-responder vaccinees (132/144) needed a single booster dose to reach adequate antibody levels. The administration of a booster dose of PCEC rabies vaccine did not raise any safety concerns. The PCEC rabies vaccine provides immunity lasting for up to 10 years regardless of the primary schedule applied or if being administered alone or concomitantly with the Japanese encephalitis vaccine.

## 1. Introduction

Rabies is a lethal viral disease that is usually transmitted through the bite of an infected animal [1] and is vaccine-preventable, before or after exposure to the virus [2]. Rabies remains an important risk, notably to travelers to endemic countries, for whom pre-exposure prophylaxis (PrEP) is recommended [3,4]. PrEP is administered according to a primary series. Booster doses can be subsequently administered every 2–5 years [5]. Post-exposure prophylaxis (PEP) should be administered without delay if a suspected exposure to rabies virus occurred [2].

The purified chick embryo cell (PCEC) rabies vaccine (*Rabipur*, Bavarian Nordic) is indicated for active immunization against rabies in individuals of all ages [5]. According to the manufacturer, three intramuscular doses of PCEC rabies vaccine are needed for PrEP in previously unvaccinated individuals [5]. They can be administered either according to a conventional regimen on days (D) 0, D7, and D21/28 [5] or according to an accelerated one-week regimen on D0, D3, and D7, in adults 18–65 years of age [5].

Due to increasing evidence, especially for intradermal application, the World Health Organization (WHO) Expert Report published in 2018 [2] recommended a shortened two-dose PrEP regimen on D0 and D7, and no need for regular booster vaccinations. Since then, the shortened regimen for selected cell culture vaccines and embryonated egg-based vaccines has been approved by some national authorities with reference to the WHO [6–8]. In 2023, the manufacturer extended the recommendations to two intramuscular doses of PCEC rabies vaccine, administered at D0 and D7 as an alternative posology for immunocompetent individuals [5].

Rabies virus neutralizing antibodies (RVNAs) are used to determine the immunogenicity of rabies vaccines, although they do not directly correlate with protection [9]. An adequate response to rabies vaccination is typically defined by RVNA levels ≥0.5 international units [IU]/mL [2].

Adequate RVNA concentrations (≥0.5 IU/mL) persist for up to two years after primary vaccination with the PCEC rabies vaccine [10]. Primary immunization with the PCEC rabies vaccine also induces lifelong robust immunological memory in immunocompetent individuals [2,11]. Therefore, regular intramuscular booster administrations are generally considered only as an additional precaution for people at high risk of exposure, if the RVNA titers drop below 0.5 IU/mL [2]. The boostability of the PCEC rabies vaccine was shown to last for over 10 years after primary immunization [2,11]. However, more information on the RVNA persistence and boostability of the PCEC rabies vaccine administered according to the accelerated three-dose schedule (D0, D3, and D7) concomitantly with other vaccines is needed.

In a phase III randomized clinical trial (NCT01662440; parent study) we showed that immune responses to the PCEC rabies vaccine co-administered with an inactivated Vero cell-derived Japanese encephalitis (JE) vaccine (*IXIARO*, Valneva) according to an accelerated one-week three-dose regimen (D0, D3, and D7) were non-inferior to the conventional four-week regimen (D0, D7, and D28) up to one year post-vaccination [9,12].

In the current extension of the parent study (NCT02545517), we aimed to evaluate the long-term persistence of RVNA responses in participants who received different primary series of rabies PrEP vaccination regimens and to evaluate the antibody responses to PCEC rabies vaccine booster dose(s) administered to participants with RVNA concentrations <0.5 IU/mL detected during the extension study.

**Fig 1:**
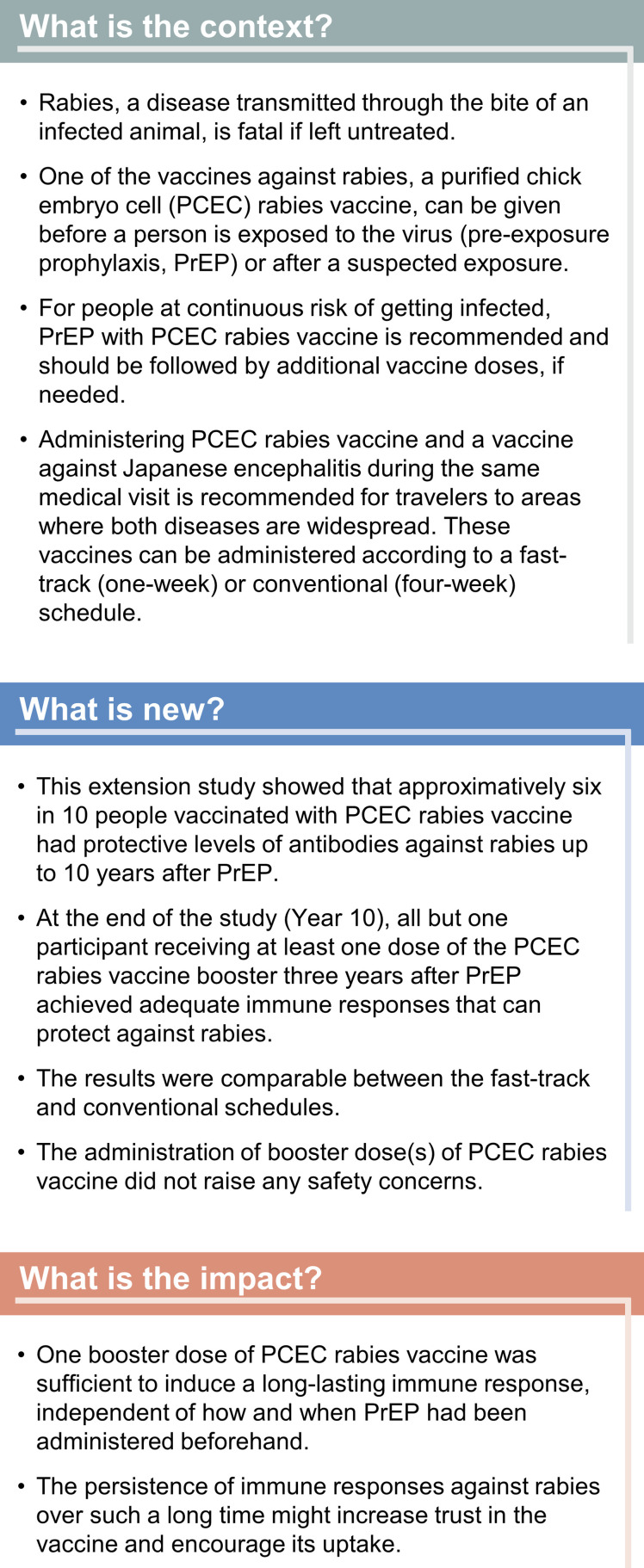
Plain language summary.

A plain language summary is presented in Fig 1.

## 2. Methods

### 2.1. Ethics statement

This study was conducted in accordance with the principles of the International Conference on Harmonization Guideline for Good Clinical Practice, the Declaration of Helsinki, and all applicable regulatory requirements. The study protocol and any subsequent amendments were approved by the Independent Ethics Committee at each participating site (Kantonale Ethikkommission, Zürich, Switzerland; Ethik-Kommission Medical University of Vienna, Vienna, Austria; Ethikkommission der Medizinischen Fakultät der LMU München, Munich, Germany). The trial is registered on ClinicalTrials.gov (NCT02545517).

### 2.2. Study design and participants

**Fig 2:**
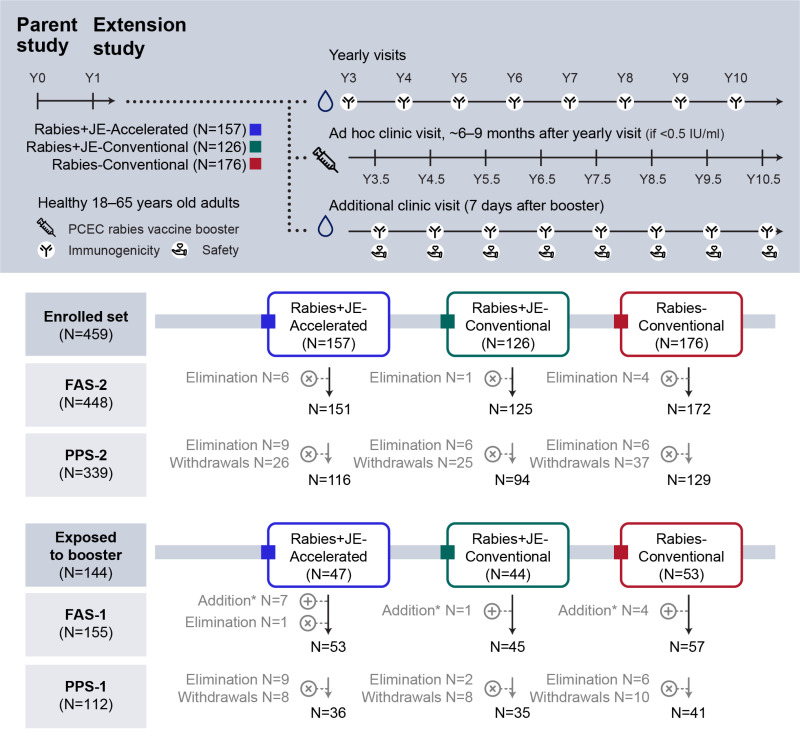
Study design and participant flowchart. *Twelve participants who received a PCEC rabies vaccine booster dose after the completion of the parent study [12] and before entering this extension study were included in the post-booster immunogenicity analysis. For these participants, the rabies virus neutralizing antibody concentrations were not tested before the administration of the booster dose. FAS-2 includes all participants who provided immunogenicity data and PPS-2 includes all participants in FAS-2 who had no major protocol deviations leading to exclusion. FAS-1 includes all participants who received at least one booster dose during the trial and provided immunogenicity data and PPS-1 includes all participants in the FAS-1 who had no major protocol deviations leading to exclusion. Rabies+JE-Accelerated, participants who received rabies vaccine concomitantly with Japanese encephalitis vaccine according to the accelerated one-week schedule; Rabies+JE-Conventional, participants who received rabies vaccine concomitantly with Japanese encephalitis vaccine according to the conventional four-week schedule; Rabies-Conventional, participants who received rabies vaccine alone according to the conventional four-week schedule; Y, year; IU, international units; PCEC, purified chick embryo cell; N, number of participants; FAS, full analysis set; PPS, per-protocol set.

This phase III, open-label study was conducted at five centers in Germany, one center in Austria, and one center in Switzerland, between 2015 and 2022, as an extension of the parent study where healthy 18–65 years old adults were enrolled [9]. Participants had been randomized 3:4:4:1 into four vaccine groups to receive three doses of the PCEC rabies vaccine and two doses of the JE vaccine either concomitantly according to the accelerated one-week schedule (Rabies+JE-Accelerated) or to the conventional four-week schedule (Rabies+JE-Conventional), or individually according to the conventional four-week schedule (PCEC rabies vaccine alone [Rabies-Conventional] or JE vaccine alone [JE-Conventional]) (Fig 2).

Those participants who had received the full rabies PrEP regimen (i.e., Rabies+JE-Accelerated, Rabies+JE-Conventional, Rabies-Conventional groups), and completed the parent study [12] without major protocol deviations were eligible for enrollment in the extension study. Each participant provided written informed consent prior to any study-related procedures. Female participants of childbearing potential were asked to practice adequate contraception for at least six months after the PCEC rabies booster vaccination. Full inclusion and exclusion criteria of the extension study are presented in S1 Text.

Each participant was followed up for approximately 10 years after completion of the primary series of accelerated or conventional rabies PrEP regimen (Fig 2).

### 2.3. Study objectives

The primary objectives of the extension study were (i) to compare the long-term (up to 10 years) persistence of RVNA responses (i.e., time until RVNA concentrations <0.5 IU/mL) in participants who received a primary series of accelerated or conventional rabies PrEP regimen in the parent study [12], (ii) to evaluate the RVNA responses to a booster dose of PCEC rabies vaccine administered to participants with RVNA concentrations <0.5 IU/mL, and (iii) to evaluate the safety of PCEC rabies vaccine booster dose(s) in participants who received a primary series of accelerated or conventional rabies PrEP regimen in the parent study [12].

The secondary objective was to describe in detail the long-term (up to 10 years) immunogenicity of the PCEC rabies vaccine in participants who received a primary series of accelerated or conventional rabies PrEP regimen in the parent study [12].

### 2.4. Study procedures

The participants’ immune responses were evaluated at yearly visits, starting from year (Y) 3. Blood samples (approximately 7 mL/visit) were drawn for RVNA concentration assessment. RVNA concentrations were measured by rapid fluorescent focus inhibition test assay [13,14] at Kansas State Veterinary Diagnostic Laboratory, Kansas, United States [15] throughout the entire study period. The rapid fluorescent focus inhibition test assay is considered a “gold standard” assay for measuring correlates of protection against rabies (RVNA) in vitro [13].

Participants with RVNA concentrations <0.5 IU/mL detected at the yearly visit were eligible to receive 1.0-mL PCEC rabies vaccine booster dose(s). The booster was administered intramuscularly in the deltoid area of the nondominant arm by designated and trained site personnel during an ad hoc clinic visit (~6–9 months after the yearly visit). Participants who had received the PCEC rabies vaccine booster dose were evaluated for post-booster immune responses via blood samples (approximately 7 mL/visit) drawn during an additional clinic visit (occurring seven days post-booster administration) and at the next yearly evaluation. The time elapsed between the booster and the next yearly evaluation was expected to be between 6–9 months. Those not reaching an adequate RVNA concentration (i.e., ≥ 0.5 IU/mL) at two consecutive determinations (seven days post-booster administration and at the next yearly evaluation) were allowed to receive an additional PCEC rabies vaccine booster dose at a following ad hoc clinic visit. If the participant was still a non-responder, subsequent management was at the discretion of the investigator.

Only serious adverse events (SAEs) following PCEC rabies vaccine booster administration were to be collected together with the associated concomitant medications, from the time of booster administration until the completion of the safety follow-up period (i.e., the day of the next yearly scheduled visit post-booster administration). SAEs occurring during the conduct of the study were classified by the investigator as probably, possibly, or not related to vaccination.

### 2.5. Statistical analyses

Up to 578 participants who had successfully completed the rabies PrEP regimens in the parent study [12] were eligible for enrollment in this extension study. Since no formal hypotheses had to be tested, sample size calculations were not computed.

Kaplan-Meier curves were produced to compare the long-term persistence of RVNA responses in participants who had received a complete rabies PrEP regimen in the parent study [12]. For each participant, time to first RVNA concentrations <0.5 IU/mL was calculated. Kaplan-Meier estimates of the survival function along with 95% confidence intervals (CIs) were computed and displayed per vaccination regimen. Group comparisons were performed using the Cox proportional hazard regression model; hazard ratios were computed with 95% CIs.

All RVNA geometric mean concentrations (GMCs) were based on the logarithmically transformed (log_10_) values. Yearly GMCs, with the associated 95% CIs were computed for each vaccine group by taking the exponential of the corresponding log-transformed (least squares) means and 95% CIs, from an analysis of variance model with fixed factors for group and center. Group differences along with 95% CIs were also computed. Participants were included in this analysis until they received the PCEC rabies vaccine booster and were excluded thereafter.

For the participants receiving a PCEC rabies vaccine booster dose, boostability was analyzed seven days post-booster administration and at the next yearly visit post-booster administration by providing geometric mean ratios and associated 95% CIs, considering the RVNA concentration measured before the booster visit (i.e., previous yearly visit) as baseline (denominator) and the RVNA concentration measured seven days post-booster administration and at the next yearly evaluation as the numerator.

The pooled RVNA concentrations of all boosted participants were analyzed using an analysis of variance model that accounted for vaccine regimen group, timing of the booster dose, and their interaction.

To check the long-term immunogenicity in participants who received a primary series of PCEC rabies vaccine in the parent study, GMCs as well as percentages of participants with RVNA concentrations ≥0.5 IU/mL (measured by rapid fluorescent focus inhibition test assay) were measured yearly, from Y3 to Y10.

For those participants receiving more than one PCEC rabies vaccine booster dose during the study, subsequent RVNA values after the second booster dose were set to half of the detection level (i.e., 0.1 IU/mL) until study end.

Immunogenicity analyses were performed both on the full analysis set (FAS) and per-protocol set (PPS). Booster immunogenicity analyses were performed on FAS-1, including all participants who received at least one booster dose during the trial and provided immunogenicity data, and on the PPS-1, including all participants in the FAS-1 who had no major protocol deviations leading to exclusion. Long-term immunogenicity analyses were performed on the FAS-2, including all participants who provided immunogenicity data, and PPS-2, including all participants in FAS-2 who had no major protocol deviations leading to exclusion.

Safety analyses were performed on the safety set, including all participants who were exposed to booster dose and reported safety data.

All analyses were performed using SAS Software version 9.2 or higher.

## 3. Results

### 3.1. Demographics and disposition of participants

Out of 459 enrolled participants, 356 (77.6%) completed the study at Y10. One hundred three (22.4%) participants withdrew from the study with lost to follow-up as the main reason for study discontinuation (n = 44, 9.6%). Eleven to 14 participants withdrew from the extension study after every yearly visit, except for the seventh (last) yearly visit. After the seventh yearly visit, 26 participants (25.2%) prematurely discontinued the study; 11 of them were lost to follow-up.

The mean age at enrollment in the parent study was 37.7 (±13.0) years. Participants’ demographic and baseline characteristics are presented in Table 1.

**Table 1:** Characteristics of the participants at baseline (i.e., at year 3 after primary vaccination; enrolled set).

Characteristic	Rabies+JE-Accelerated	Rabies+JE-Conventional	Rabies-Conventional	Total
	N = 157	N = 126	N = 176	N = 459
**Age at enrollment** (years); mean±SD	38.7 ± 13.0	37.9 ± 13.2	36.6 ± 12.8	37.7 ± 13.0
**Gender**; n (%)				
Female	94 (59.9)	61 (48.4)	101 (57.4)	256 (55.8)
Male	63 (40.1)	65 (51.6)	75 (42.6)	203 (44.2)
**Country**; n (%)				
Austria	27 (17.2)	16 (12.7)	24 (13.6)	67 (14.6)
Germany	112 (71.3)	90 (71.4)	125 (71.0)	327 (71.2)
Switzerland	18 (11.5)	20 (15.9)	27 (15.3)	65 (14.2)
**Race**; n (%)				
White	154 (98.1)	125 (99.2)	173 (98.3)	452 (98.5)
Asian	0 (0.0)	1 (0.8)	1 (0.6)	2 (0.4)
Black or African American	2 (1.3)	0 (0.0)	0 (0.0)	2 (0.4)
Other	1 (0.6)	0 (0.0)	2 (1.1)	3 (0.7)
**BMI** (kg/m^2^); mean±SD	25.6 ± 5.2	25.1 ± 4.6	25.3 ± 4.5	25.3 ± 4.8

Rabies+JE-Accelerated, participants who received rabies vaccine concomitantly with Japanese encephalitis vaccine according to the accelerated one-week schedule; Rabies+JE-Conventional, participants who received rabies vaccine concomitantly with Japanese encephalitis vaccine according to the conventional four-week schedule; Rabies-Conventional, participants who received rabies vaccine alone according to the conventional four-week schedule; N, total number of participants for each study group; SD, standard deviation; n (%), number (percentage) of participants in a given category; BMI, body mass index.

Overall, 144 participants received at least one PCEC rabies vaccine booster dose during the study (S1 Table). Of these, 143 participants provided blood samples and were included in FAS-1. Before the study start, an additional 12 participants were administered a PCEC rabies vaccine booster dose (i.e., between the end of the parent study and the beginning of the current extension study) and had no pre-booster immunogenicity data collected. These 12 participants were also included in FAS-1. One hundred twelve participants were included in PPS-1 (Fig 2).

A total of 448 participants were included in FAS-2 for long-term persistence of PCEC rabies vaccine immune responses, of whom 339 were included in PPS-2 (Fig 2).

### 3.2. Immunogenicity

#### 3.2.1. Long-term persistence of immune seroresponses.

**Fig 3:**
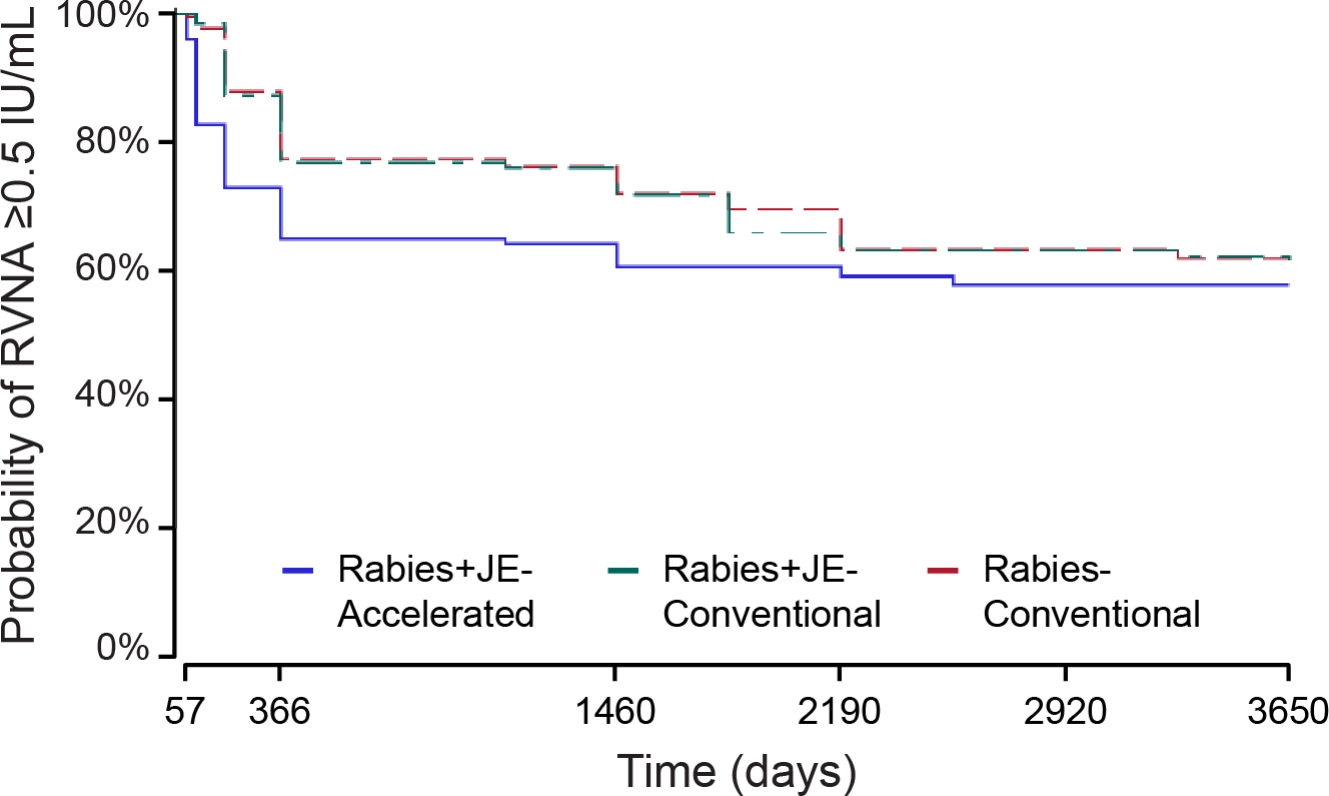
Kaplan-Meier estimation of the survival function of RVNAs (full analysis set 2). RVNA, rabies virus neutralizing antibody; IU, international units; Rabies+JE-Accelerated, participants who received rabies vaccine concomitantly with Japanese encephalitis vaccine according to the accelerated one-week schedule; Rabies+JE-Conventional, participants who received rabies vaccine concomitantly with Japanese encephalitis vaccine according to the conventional four-week schedule; Rabies-Conventional, participants who received rabies vaccine alone according to the conventional four-week schedule.

**Fig 4:**
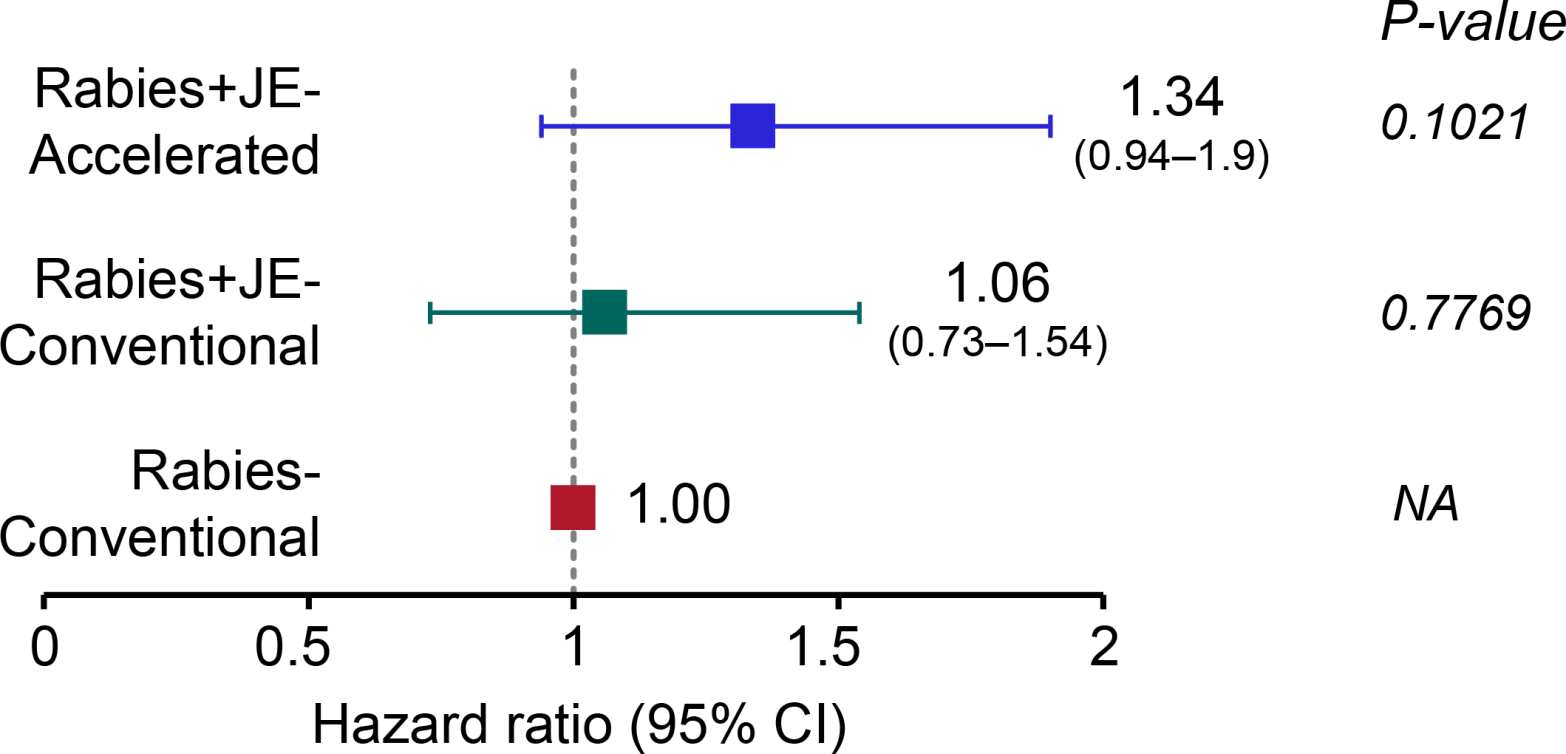
Adjusted hazard ratio for time to first RVNA concentration below 0.5 IU/mL (full analysis set 2). RVNA, rabies virus neutralizing antibody; IU, international units; Rabies+JE-Accelerated, participants who received rabies vaccine concomitantly with Japanese encephalitis vaccine according to the accelerated one-week schedule; Rabies+JE-Conventional, participants who received rabies vaccine concomitantly with Japanese encephalitis vaccine according to the conventional four-week schedule; Rabies-Conventional, participants who received rabies vaccine alone according to the conventional four-week schedule; NA, not applicable; 95% CI, 95% confidence interval.

The Kaplan-Meier curve for the accelerated schedule (Rabies+JE-Accelerated) showed a quicker drop in RVNA concentration as compared to the other two conventional groups’ curves (Rabies+JE-Conventional, Rabies-Conventional), for both FAS-2 (Fig 3) and PPS-2 (Fig A in S2 Text). However, the adjusted hazard ratios (aHRs) for the comparison between the accelerated (Rabies+JE-Accelerated) and the conventional (Rabies+JE-Conventional) schedule did not reach statistical significance (p < 0.05), with aHRs of 1.34 (95% CI: 0.94–1.90, p = 0.1021) and 1.47 (95% CI: 0.98–2.22, p = 0.0620) in the FAS-2 and PPS-2, respectively (Fig 4 and S2 Table).

**Fig 5:**
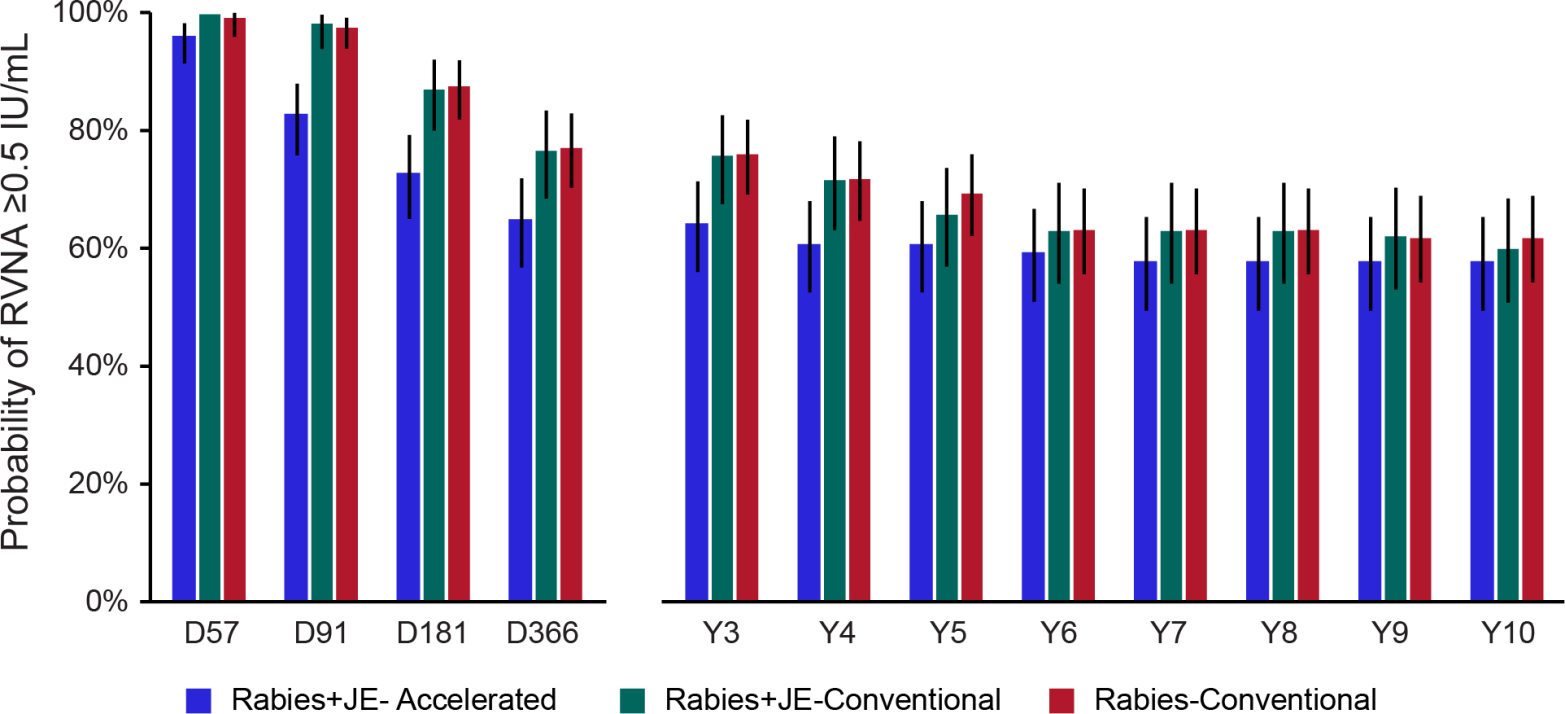
Probability of long-term RVNA persistence (full analysis set 2). RVNA, rabies virus neutralizing antibody; IU, international units; Rabies+JE-Accelerated, participants who received rabies vaccine concomitantly with Japanese encephalitis vaccine according to the accelerated one-week schedule; Rabies+JE-Conventional, participants who received rabies vaccine concomitantly with Japanese encephalitis vaccine according to the conventional four-week schedule; Rabies-Conventional, participants who received rabies vaccine alone according to the conventional four-week schedule; D, day; Y, year. Note: Error bars represent 95% confidence intervals.

At the end of the study (Y10), the probability of attaining an RVNA concentration ≥0.5 IU/mL was 57.8% (95% CI: 49.4–65.3) in the Rabies+JE-Accelerated group, 60.2% (95% CI: 50.8–68.4) in the Rabies+JE-Conventional group, and 62.0% (95% CI: 54.2–68.9) in the Rabies-Conventional group (Fig 5). A similar trend was observed in the PPS-2 (S3 Table).

Yearly GMCs were computed to further describe the long-term immunogenicity as part of the secondary objective. At Y10, the measured GMC values for participants in FAS-2 were 0.72 IU/mL, 0.59 IU/mL, and 0.68 IU/mL in Rabies+JE-Accelerated, Rabies+JE-Conventional, and Rabies-Conventional schedule groups, respectively (Table 2).

**Table 2:** Long-term RVNA persistence using repeated measures (full analysis set 2).

Timepoint	GMC (IU/mL; 95% CI)			
	Rabies+JE-Accelerated	Rabies+JE-Conventional	Rabies-Conventional	Pooled regimens
Year 3	1.16 (0.87–1.55)	1.21 (0.88–1.65)	1.30 (0.99–1.70)	1.22 (1.03–1.45)
Year 4	0.73 (0.55–0.97)	0.69 (0.50–0.94)	0.82 (0.63–1.07)	0.74 (0.62–0.88)
Year 5	0.70 (0.53–0.94)	0.66 (0.48–0.90)	0.74 (0.56–0.96)	0.70 (0.59–0.83)
Year 6	0.60 (0.45–0.80)	0.50 (0.37–0.69)	0.58 (0.44–0.76)	0.56 (0.47–0.66)
Year 7	0.60 (0.45–0.79)	0.49 (0.36–0.67)	0.61 (0.47–0.80)	0.56 (0.47–0.67)
Year 8	0.76 (0.57–1.01)	0.56 (0.41–0.77)	0.68 (0.52–0.89)	0.66 (0.56–0.79)
Year 9	0.70 (0.53–0.94)	0.53 (0.39–0.73)	0.62 (0.47–0.81)	0.61 (0.52–0.73)
Year 10	0.72 (0.54–0.96)	0.59 (0.43–0.81)	0.68 (0.52–0.89)	0.66 (0.56–0.79)

RVNA, rabies virus neutralizing antibody; GMC, geometric mean concentration; IU, international units; 95% CI, 95% confidence interval; Rabies+JE-Accelerated, participants who received rabies vaccine concomitantly with Japanese encephalitis vaccine according to the accelerated one-week schedule; Rabies+JE-Conventional, participants who received rabies vaccine concomitantly with Japanese encephalitis vaccine according to the conventional four-week schedule; Rabies-Conventional, participants who received rabies vaccine alone according to the conventional four-week schedule.

#### 3.2.2. Boostability of PCEC rabies vaccine.

Of the 459 enrolled participants, 315 received no PCEC rabies vaccine booster (68.6%) and 144 (31.4%) received at least one PCEC rabies vaccine booster dose during the 10-year follow-up period. Of the 144 boosted participants, 132 received exactly one PCEC rabies vaccine booster dose. Eight participants in the Rabies+JE-Accelerated group, one participant in the Rabies+JE-Conventional group, and three participants in the Rabies-Conventional group received multiple (i.e., 2–7) PCEC rabies virus booster doses throughout the study (12/144, 8.3%; S1 Table). One participant in the Rabies-Conventional group received a booster each year. The RVNA concentrations for this participant were <0.1 IU/mL after primary vaccination with the PCEC rabies vaccine in the parent study and remained <0.1 IU/mL throughout the entire follow-up period of the extension study.

**Fig 6:**
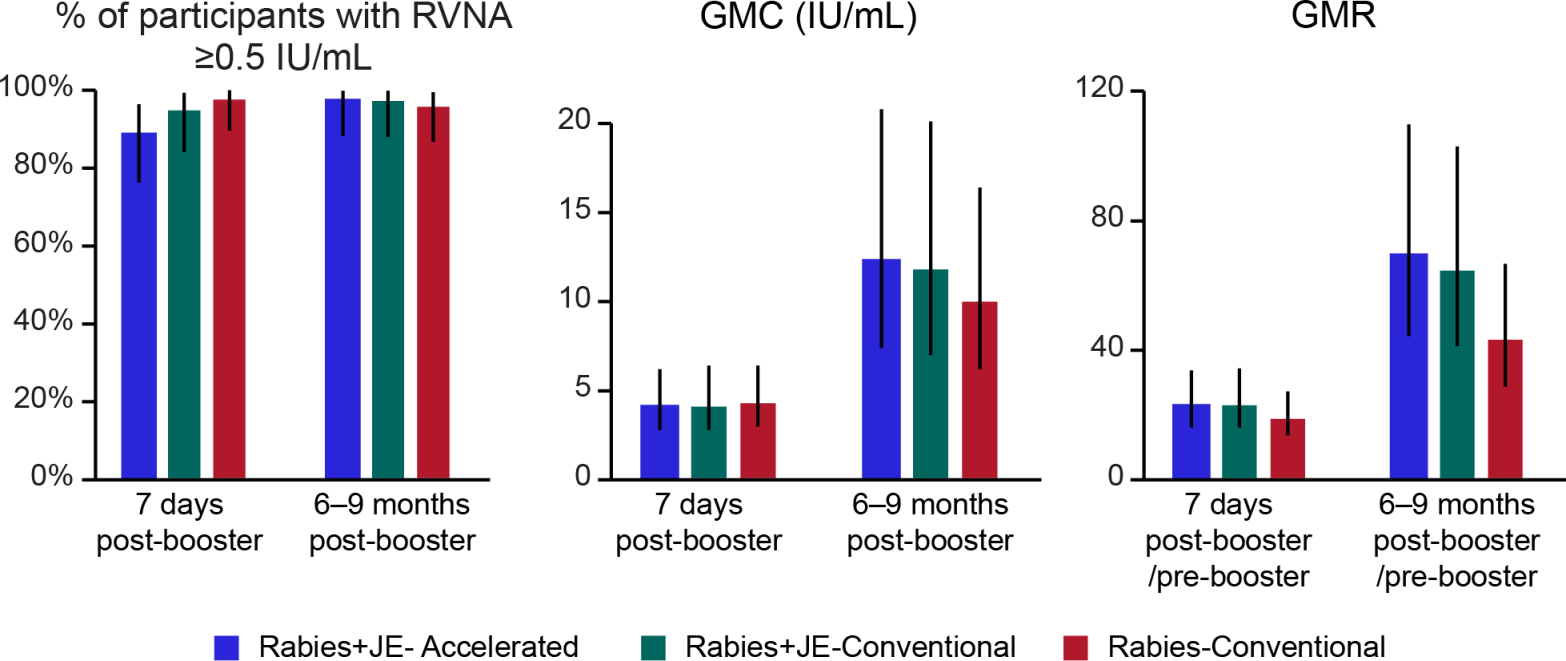
Overall (Y3–Y10) boostability of the PCEC rabies vaccine (full analysis set 1). Y, year; PCEC, purified chick embryo cell; RVNA, rabies virus neutralizing antibody; IU, international units; GMC, geometric mean concentration; GMR, geometric mean ratio; Rabies+JE-Accelerated, participants who received rabies vaccine concomitantly with Japanese encephalitis vaccine according to the accelerated one-week schedule; Rabies+JE-Conventional, participants who received rabies vaccine concomitantly with Japanese encephalitis vaccine according to the conventional four-week schedule; Rabies-Conventional, participants who received rabies vaccine alone according to the conventional four-week schedule. Note: Error bars represent 95% confidence intervals.

Irrespectively of the time passed since the PCEC rabies vaccine booster dose was administered, the number and percentage of participants with RVNA concentration ≥0.5 IU/mL measured seven days post-booster dose administration and at the next yearly scheduled visit were similar across the three schedule groups in FAS-1 (Fig 6 and S4 Table). Seven days after the PCEC rabies vaccine booster dose administration, RVNA concentration was ≥ 0.5 IU/mL for 89.1%, 95.3%, and 98.1% of participants in Rabies+JE-Accelerated, Rabies+JE-Conventional, and Rabies-Conventional schedule groups, respectively. After 6–9 months post-booster administration, RVNA concentration was ≥ 0.5 IU/mL for 97.8%, 97.7%, and 96.2% of participants in Rabies+JE-Accelerated, Rabies+JE-Conventional, and Rabies-Conventional schedule groups, respectively. GMCs seven days post-booster administration were 4.2 IU/mL, 4.2 IU/mL, and 4.4 IU/mL for participants in Rabies+JE-Accelerated, Rabies+JE-Conventional, and Rabies-Conventional schedule groups, respectively. After 6–9 months post-booster administration, GMCs increased across all study groups. An increase was also observed for geometric mean ratios (Fig 6 and S4 Table). Similar results were found for PPS-1 (S5 Table).

For those participants included in FAS-1 with available persistence data up to seven years post-booster administration, the GMCs seven years after receiving the PCEC rabies vaccine booster dose were 4.30 IU/mL (95% CI: 2.41–7.67), 3.86 IU/mL (95% CI: 2.05–7.26), 4.04 IU/mL (95% CI: 2.28–7.17), and 4.06 IU/mL (2.82–5.86) in Rabies+JE-Accelerated, Rabies+JE-Conventional, Rabies-Conventional schedule groups, and for the pooled regimens, respectively (S6 Table).

### 3.3. Safety

Overall, during the extension study safety follow-up, four participants (2.8%) reported SAEs. Among three participants (5.7%) in the Rabies-Conventional group, one reported diarrhea of severe intensity (grade 3), another reported leiomyoma, and a third committed suicide. Deep vein thrombosis and pulmonary embolism were both reported in one participant (2.3%) in the Rabies+JE-Conventional group. No participant in the Rabies+JE-Accelerated group reported any SAE. None of the SAEs were considered by the investigator as related to the study vaccine.

## 4. Discussion

This is the first long-term study to investigate the requirement for and the timing of a booster administration following an accelerated three-dose PrEP regimen of PCEC rabies vaccine administered concomitantly with the JE vaccine. PCEC rabies vaccine boosters are recommended by the manufacturer every 2–5 years in case of ongoing risk [5]; however, guidance by the WHO only recommends them for those at high risk of exposure [16]. Our study shows that the PCEC rabies vaccine provides long-term immunity and boostability also when administered concomitantly with the JE vaccine, for all tested schedules without significant differences, and supports the WHO recommendations regarding booster administration [2].

The statistical analyses carried out approximately 10 years after completion of the primary series of accelerated or conventional rabies PrEP regimen showed a similar persistence of immunogenicity in the three study groups. Similar results were found in the parent study, where the non-inferiority of the RVNA response was demonstrated for all three vaccination regimens [9,12]. Even though a faster decrease in RVNA persistence for the Rabies+JE-Accelerated group compared to the conventional regimens (Rabies+JE-Conventional, Rabies-Conventional) was observed, it did not reach statistical significance when comparing the aHRs.

In the parent study, short-term immunogenicity analysis showed that 97% participants in the Rabies+JE-Accelerated group had adequate RVNA concentrations (≥0.5 IU/mL) 57 days post-primary immunization, compared to 99% participants in the Rabies+JE-Conventional and 100% participants in the Rabies-Conventional groups [9]. At D366, 68% participants in the Rabies+JE-Accelerated group had RVNA concentrations ≥0.5 IU/mL, compared to 76% in the Rabies+JE-Conventional group and 80% in the Rabies-Conventional group [12]. This trend was maintained in the extension study; at Y10, 57.8% participants in the Rabies+JE-Accelerated group had RVNA concentrations ≥0.5 IU/mL, compared to 60.2% participants in the Rabies+JE-Conventional and 62.0% participants in the Rabies-Conventional groups.

Several studies have found that PrEP with rabies vaccines (administered both intradermally and intramuscularly) elicits long-lasting antibody responses in adults for up to 10 years [11,17–22]. Our results are in line with these previous findings; 68.6% of the enrolled participants in the current study did not require any booster dose as RVNA levels ≥0.5 IU/mL were detected 10 years after the primary vaccination. The sustained immunogenicity of the PCEC rabies vaccine is confirmed by our results, even if co-administered with the JE vaccine.

The administration of ≥1 booster dose(s) several years after PrEP with rabies vaccines (intradermally and intramuscularly) was previously shown to rapidly reactivate the immunological memory [19–21,23]. Throughout the follow-up period of the present extension study, of the participants attending the seven-day post-booster administration visit, 89.1%–98.1% across study groups had RVNA concentrations ≥0.5 IU/mL. The percentage of participants expressing adequate RVNA levels at 6–9 months post-booster administration was 96.2%–97.8% across groups. In all groups, GMCs at seven days post-booster administration were ≥19-fold higher than baseline levels and continued to increase overtime, being ≥43-fold higher than baseline levels at 6–9 months post-booster administration. This confirms the high boostability of the PCEC rabies vaccine even when co-administered with the JE vaccine in the primary schedule. The percentage of participants with adequate RVNA levels following administration of the booster dose was also similar between timepoints from Y3 to Y10, indicating that immunological memory is reliably reactivated at different intervals, over extended periods following administration of various primary regimens.

In this extension study, 3% of all participants were defined as low/non-responder, i.e., in 12 persons RVNA concentrations declined considerably after receiving a PCEC rabies vaccine booster dose; therefore, multiple booster doses (i.e., 2–7) were administered. The low/non-responsiveness rate to any vaccine is estimated at 2%–10% [24]. The frequency of low/non-responder vaccinees in this extension study (12/144, 8.3%) did not exceed the expected rates.

In this study, we had one non-responder vaccinee in the Rabies-Conventional group. The decision to administer seven PCEC rabies vaccine booster doses to this participant was at the investigator’s discretion. The reasons for failing to mount an immune response were not explored further. However, this individual was considered healthy, with no serious medical history, and did not meet any of the study’s exclusion criteria. In such non-responders, the administration of rabies immunoglobulins alongside PEP should be considered. However, response evaluation in real-life settings is not standard practice, and therefore non-responders are not readily identified. The use of PCEC vaccination and rabies immunoglobulins, according to standard medical practice, increases the chance of protection after exposure in potential non-responders.

Our study found no safety concerns following the administration of the PCEC rabies vaccine booster. The number of participants who experienced at least one SAE was similar in both the extension (n = 4) and parent study (n = 7). However, compared to the parent study where two participants had SAEs that were considered probably/possibly related to the study vaccine [9], none of the SAEs reported in this extension study were considered related to the administration of the PCEC rabies vaccine booster.

There is evidence that cell culture and embryonated egg-based rabies vaccines can be co-administered with JE vaccines, measles-mumps-rubella vaccines, diphtheria-tetanus-pertussis vaccines, and poliomyelitis vaccines without negatively impacting the safety and immunogenicity of the rabies vaccine [1,11]. Co-administration of vaccines against rabies and JE were previously shown to be safe and immunogenic for approximatively five years [11]. Our results have shown that the JE vaccine does not have an impact on the persistence of RVNA concentrations over the course of 10 years.

The strength of this study is the high retention rate of the large, enrolled population considering the prolonged duration of the study. Furthermore, this is the first study to evaluate the long-term persistence of RVNA concentrations induced by different PCEC rabies vaccine PrEP priming schedules.

There were a few potential study limitations that we need to consider. First, due to its design (i.e., long-term extension of a parent study), the analyses were mostly descriptive in nature. Furthermore, the results of our study cannot be generalized to the pediatric population, considering that only adult participants were allowed to be enrolled in the parent study. Finally, our results cannot be generalized to elderly populations (i.e., > 65 years of age) considering the limited number of elderly people that participated in the extension study, who might need boosters at earlier timepoints.

## 5. Conclusions

The statistical analyses carried out after the completion of the study at Y10 showed a similar immunogenicity persistence across the three study groups. We also demonstrated a strong immunogenic response to a PCEC rabies vaccine booster dose, irrespective of the primary regimen received. Furthermore, the response was independent of the time elapsed since the last primary vaccine dose administration. This indicates that all participants receiving a PCEC rabies vaccine booster dose, three or more years after the primary vaccination series, exhibited a similar response to the booster dose. These results, spanning over a decade, demonstrate the long-term immunogenicity and the boostability of the PCEC rabies vaccine, along with the safety of administering PCEC booster doses. Collectively, these data strengthen the breadth of clinical evidence related to the vaccine and support its use, particularly for travelers to rabies endemic areas.

## Supporting information

S1 Text Inclusion and exclusion criteria. (DOCX)

S2 Text Supplementary figure. Fig A: The figure presents the time to first RVNA concentration above 0.5 IU/mL by study group in the per-protocol set 2.
(DOCX)

S1 Table Number and percentage of participants receiving a booster dose during the extension study (enrolled set). (DOCX)

S2 Table Adjusted hazard ratio for time to first RVNA concentration below 0.5 IU/mL (per-protocol set 2). (DOCX)

S3 Table Probability of long-term antibody persistence (per-protocol set 2). (DOCX)

S4 Table Boostability of the PCEC rabies vaccine (full analysis set 1). (DOCX)

S5 Table Boostability of the PCEC rabies vaccine (per-protocol set 1). (DOCX)

S6 Table Antibody persistence after booster dose using repeated measures – yearly GMCs (full analysis set 1). (DOCX)

S1 File CONSORT checklist. (DOCX)
