# Supplementary material for: Long-term persistence and boostability of immune responses following different rabies pre-exposure prophylaxis priming schedules of a purified chick embryo cell rabies vaccine administered alone or concomitantly with a Japanese encephalitis vaccine
Source: PLoS Negl Trop Dis. 2025 May 27;19(5):e0013118. doi: 10.1371/journal.pntd.0013118 (PMC12136438; doi:10.1371/journal.pntd.0013118)
Supplement: S1 Text — (DOCX) [file pntd.0013118.s001.docx]

## S1 Text. Inclusion and exclusion criteria

**Inclusion criteria**

- All individuals who were randomized to accelerated rabies and JE vaccination (Rabies+JE-Accelerated), to conventional rabies and Japanese encephalitis (JE) vaccination (Rabies+JE-Conventional), or to conventional rabies groups (Rabies-Conventional) during the parent study [1], who received the full pre-exposure prophylaxis (PrEP) regimen and completed the trial following the study protocol.
- Individuals who have voluntarily given written informed consent after the nature of the study has been explained according to local regulatory requirements, prior to study entry.
- Individuals who can comply with the study procedures.
- Male or female individuals of non-childbearing potential, or females of childbearing potential who are using an effective birth control method which they intend to use for at least six months after the booster administration. This criterion is applicable only for those participants who receive a booster dose.

## Exclusion criteria

**Prior to extension study entry, each participant must not have:**

- Completed the parent study [1] without receiving the full three rabies vaccine doses following the assigned PrEP regimen.
- History of exposure to suspected or confirmed rabid animal.
- Receipt of rabies immunoglobulins, rabies post-exposure prophylaxis following completion of the parent study [1].
- Hypersensitivity, including allergy, to any component of vaccines, medicinal products, or medical equipment whose use is foreseen in this study.
- Clinical conditions representing a contraindication to intramuscular vaccination and blood draws.
- Systemic administration of corticosteroids for more than 14 consecutive days within 90 days prior to informed consent or planning to receive them during the participation to the study.
- Administration of antineoplastic and immunomodulating agents or radiotherapy within 90 days prior to informed consent or planning to receive them during the participation to the study.
- Received immunoglobulins or any blood products within 180 days prior to informed consent or planning to receive them during the participation to the study.
- Study personnel as well as their immediate family or household member.
- Any other clinical condition that, in the opinion of the investigator, might pose additional risk to the individual due to participation in the study.

**Prior to yearly visit, each participant must not have:**

- History of exposure to suspected or confirmed rabid animal.
- Receipt of rabies immunoglobulins, non-study rabies vaccine following completion of the parent study [1].
- Hypersensitivity, including allergy, to any component of vaccines, medicinal products, or medical equipment whose use is foreseen in this study.
- Clinical conditions representing a contraindication to intramuscular vaccination and blood draws.
- Systemic administration of corticosteroids for more than 14 consecutive days within 90 days prior to informed consent or planning to receive them during the participation to the study.
- Administration of antineoplastic and immunomodulating agents or radiotherapy within 90 days prior to informed consent or planning to receive them during the participation to the study.
- Received immunoglobulins or any blood products within 180 days prior to informed consent or planning to receive them during the participation to the study.
- Study personnel as well as their immediate family or household member.
- Any other clinical condition that, in the opinion of the investigator, might pose additional risk to the participant due to participation in the study.

**Prior to booster administration, each eligible participant should be in good health status and must not have:**

- Progressive, unstable, or uncontrolled clinical conditions.
- Abnormal function of the immune system resulting from:

a. Clinical conditions.

b. Systemic administration of corticosteroids for more than 14 consecutive days within 90 days prior to the booster visit, or receipt or planned receipt during the participation to the study.

c. Administration of antineoplastic and immunomodulating agents or radiotherapy within 90 days prior to the booster visit, or receipt or planned receipt during the participation to the study.

- Receipt of non-study rabies vaccine.
- Receipt of any other vaccines within 28 days prior to the booster dose or planning to receive any vaccine within 28 days from the booster dose.
- Receipt of any investigational or non-registered medicinal product within 14 days before booster dose till next yearly clinic visit after booster dose administration.
- Receipt of anti-malarial medications (e.g., mefloquine) within 14 days before booster dose until next yearly clinic visit after booster dose administration. Prior to receipt of booster dose, participants must be evaluated to confirm that they are in good health, and they are eligible for subsequent vaccination. If participants meet any of the original exclusion criteria listed above, they should not receive additional vaccinations.

**References:**

[1] Jelinek T, Cramer JP, Dieckmann S, Hatz C, Paulke-Korinek M, Alberer M, et al. Evaluation of rabies immunogenicity and tolerability following a purified chick embryo cell rabies vaccine administered concomitantly with a Japanese encephalitis vaccine. Travel Med Infect Dis. 2015;(13):241-250. https://doi.org/10.1016/j.tmaid.2015.05.008
