# Supplementary material for: Long-term persistence and boostability of immune responses following different rabies pre-exposure prophylaxis priming schedules of a purified chick embryo cell rabies vaccine administered alone or concomitantly with a Japanese encephalitis vaccine
Source: PLoS Negl Trop Dis. 2025 May 27;19(5):e0013118. doi: 10.1371/journal.pntd.0013118 (PMC12136438; doi:10.1371/journal.pntd.0013118)
Supplement: S2 Text — Fig A: The figure presents the time to first RVNA concentration above 0.5 IU/mL by study group in the per-protocol set 2. (DOCX) [file pntd.0013118.s002.docx]

## S2 Text. Supplementary figure

## Fig A. Kaplan-Meier estimation of the survival function of RVNAs (per-protocol set 2)


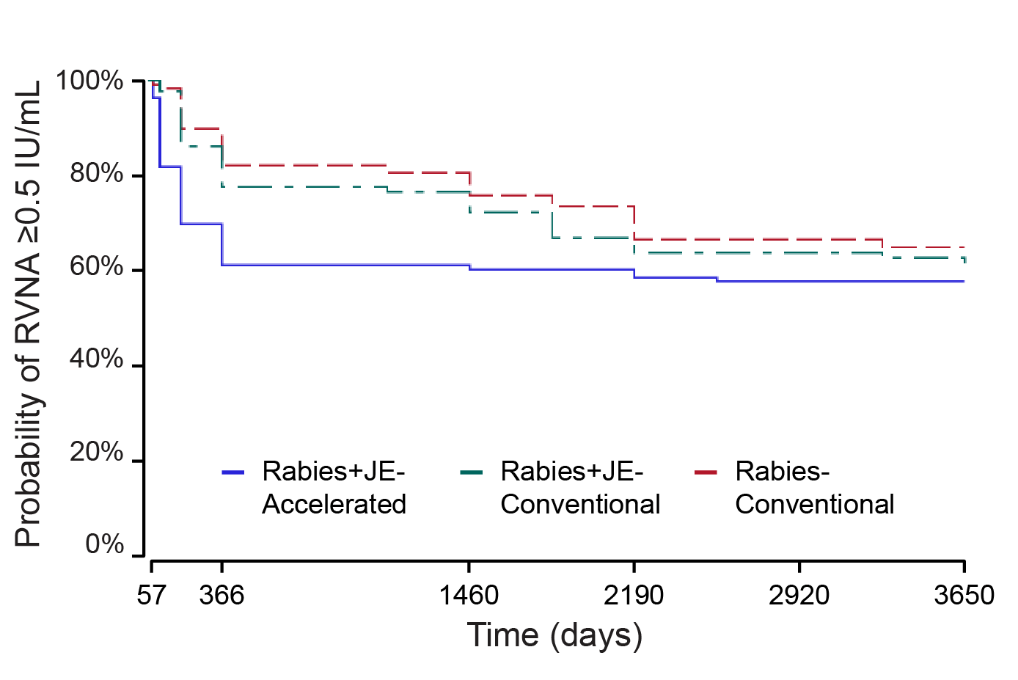


RVNA, rabies virus neutralizing antibody; IU, international units; Rabies+JE-Accelerated, participants who received rabies vaccine concomitantly with Japanese encephalitis vaccine according to the accelerated one-week schedule; Rabies+JE-Conventional, participants who received rabies vaccine concomitantly with Japanese encephalitis vaccine according to the conventional four-week schedule; Rabies-Conventional, participants who received rabies vaccine alone according to the conventional four-week schedule.
