# Supplementary material for: Long-term persistence and boostability of immune responses following different rabies pre-exposure prophylaxis priming schedules of a purified chick embryo cell rabies vaccine administered alone or concomitantly with a Japanese encephalitis vaccine
Source: PLoS Negl Trop Dis. 2025 May 27;19(5):e0013118. doi: 10.1371/journal.pntd.0013118 (PMC12136438; doi:10.1371/journal.pntd.0013118)
Supplement: S1 Table — (DOCX) [file pntd.0013118.s003.docx]

## S1 Table. Number and percentage of participants receiving a booster dose during the extension study (enrolled set)

| **Number of doses** | **n (%)** | | | |
| --- | --- | --- | --- | --- |
|  | **Rabies+JE-Accelerated** | **Rabies+JE-Conventional** | **Rabies-Conventional** | **Total** |
|  | N=157 | N=126 | N=176 | N=459 |
| No booster | 110 (70.1) | 82 (65.1) | 123 (69.9) | 315 (68.6) |
| At least 1 dose | 47 (29.9) | 44 (34.9) | 53 (30.1) | 144 (31.4) |
| Exactly 1 dose | 39 (24.8) | 43 (34.1) | 50 (28.4) | 132 (28.8) |
| Exactly 2 doses | 4 (2.5) | 1 (0.8) | 1 (0.6) | 6 (1.3) |
| Exactly 3 doses | 2 (1.3) | 0 (0.0) | 1 (0.6) | 3 (0.7) |
| Exactly 4 doses | 2 (1.3) | 0 (0.0) | 0 (0.0) | 2 (0.4) |
| Exactly 7 doses | 0 (0.0) | 0 (0.0) | 1 (0.6) | 1 (0.2) |

Rabies+JE-Accelerated, participants who received rabies vaccine concomitantly with Japanese encephalitis vaccine according to the accelerated one-week schedule; Rabies+JE-Conventional, participants who received rabies vaccine concomitantly with Japanese encephalitis vaccine according to the conventional four-week schedule; Rabies-Conventional, participants who received rabies vaccine alone according to the conventional four-week schedule; N, total number of participants for each study group; n (%), number (percentage) of participants in a given category.
