# Supplementary material for: Long-term persistence and boostability of immune responses following different rabies pre-exposure prophylaxis priming schedules of a purified chick embryo cell rabies vaccine administered alone or concomitantly with a Japanese encephalitis vaccine
Source: PLoS Negl Trop Dis. 2025 May 27;19(5):e0013118. doi: 10.1371/journal.pntd.0013118 (PMC12136438; doi:10.1371/journal.pntd.0013118)
Supplement: S2 Table — (DOCX) [file pntd.0013118.s004.docx]

## S2 Table. Adjusted hazard ratio for time to first RVNA concentration below 0.5 IU/mL (per-protocol set 2)

|  | **Rabies+JE-Accelerated** | **Rabies+JE-Conventional** | **Rabies-Conventional** |
| --- | --- | --- | --- |
| Hazard ratio (95% CI) | 1.47 (0.98–2.22) | 1.17 (0.76–1.81) | 1.00 |
| P-value | 0.0620 | 0.4830 | - |

RVNA, rabies virus neutralizing antibody; IU, International units; Rabies+JE-Accelerated, participants who received rabies vaccine concomitantly with Japanese encephalitis vaccine according to the accelerated one-week schedule; Rabies+JE-Conventional, participants who received rabies vaccine concomitantly with Japanese encephalitis vaccine according to the conventional four-week schedule; Rabies-Conventional, participants who received rabies vaccine alone according to the conventional four-week schedule; 95% CI, 95% confidence interval.
