# Supplementary material for: Long-term persistence and boostability of immune responses following different rabies pre-exposure prophylaxis priming schedules of a purified chick embryo cell rabies vaccine administered alone or concomitantly with a Japanese encephalitis vaccine
Source: PLoS Negl Trop Dis. 2025 May 27;19(5):e0013118. doi: 10.1371/journal.pntd.0013118 (PMC12136438; doi:10.1371/journal.pntd.0013118)
Supplement: S3 Table — (DOCX) [file pntd.0013118.s005.docx]

## S3 Table. Probability of long-term antibody persistence (per-protocol set 2)

| **Timepoint** | **Rabies+JE-Accelerated** | | | **Rabies+JE-Conventional** | | | **Rabies-Conventional** | | |
| --- | --- | --- | --- | --- | --- | --- | --- | --- | --- |
|  | **N** | **n** | **Probability of RVNA ≥0.5 IU/mL**  **(%, 95% CI)** | **N** | **n** | **Probability of RVNA ≥0.5 IU/mL**  **(%, 95% CI)** | **N** | **n** | **Probability of RVNA ≥0.5 IU/mL**  **(%, 95% CI)** |
| Day 57 | 116 | 4 | 96.6 (91.1–98.7) | 94 | 0 | 100 | 129 | 1 | 99.2 (94.6–99.9) |
| Day 91 | 112 | 17 | 81.9 (73.6–87.8) | 94 | 2 | 97.9 (91.8–99.5) | 128 | 1 | 98.4 (93.9–99.6) |
| Day 181 | 95 | 14 | 69.8 (60.6–77.3) | 92 | 11 | 86.2 (77.4–91.7) | 127 | 11 | 89.9 (83.3–94.0) |
| Day 366 | 81 | 10 | 61.2 (51.7–69.4) | 81 | 8 | 77.7 (67.8–84.8) | 116 | 10 | 82.2 (74.4–87.8) |
| Year 3 | 71 | 1 | 61.2 (51.7–69.4) | 73 | 1 | 76.6 (66.7–83.9) | 106 | 2 | 80.6 (72.7–86.5) |
| Year 4 | 71 | 1 | 60.3 (50.8–68.6) | 72 | 4 | 72.3 (62.1–80.2) | 104 | 6 | 76.0 (67.6–82.4) |
| Year 5 | 70 | 2 | 60.3 (50.8–68.6) | 68 | 5 | 67.0 (56.5–75.5) | 98 | 3 | 73.6 (65.1–80.4) |
| Year 6 | 70 | 2 | 58.6 (49.1–67.0) | 63 | 3 | 63.8 (53.3–72.6) | 95 | 9 | 66.7 (57.8–74.1) |
| Year 7 | 68 | 1 | 57.8 (48.2–66.1) | 60 | 1 | 63.8 (53.3–72.6) | 86 | 2 | 66.7 (57.8–74.1) |
| Year 8 | 67 | 0 | 57.8 (48.2–66.1) | 60 | 1 | 63.8 (53.3–72.6) | 86 | 2 | 66.7 (57.8–74.1) |
| Year 9 | 67 | 0 | 57.8 (48.2–66.1) | 60 | 1 | 62.8 (52.2–71.6) | 86 | 2 | 65.1 (56.2–72.6) |
| Year 10 | 67 | 0 | 57.8 (48.2–66.1) | 59 | 2 | 60.6 (50.0–69.7) | 84 | 0 | 65.1 (56.2–72.6) |

Rabies+JE-Accelerated, participants who received rabies vaccine concomitantly with Japanese encephalitis vaccine according to the accelerated one-week schedule; Rabies+JE-Conventional, participants who received rabies vaccine concomitantly with Japanese encephalitis vaccine according to the conventional four-week schedule; Rabies-Conventional, participants who received rabies vaccine alone according to the conventional four-week schedule; N, number of participants still at risk; n, number of events; RVNA, rabies virus neutralizing antibody; IU, international units; 95% CI, 95% confidence interval.
