# Supplementary material for: Long-term persistence and boostability of immune responses following different rabies pre-exposure prophylaxis priming schedules of a purified chick embryo cell rabies vaccine administered alone or concomitantly with a Japanese encephalitis vaccine
Source: PLoS Negl Trop Dis. 2025 May 27;19(5):e0013118. doi: 10.1371/journal.pntd.0013118 (PMC12136438; doi:10.1371/journal.pntd.0013118)
Supplement: S4 Table — (DOCX) [file pntd.0013118.s006.docx]

## S4 Table. Boostability of the PCEC rabies vaccine (full analysis set 1)

| **Timepoint** | **Rabies+JE-Accelerated** | | | **Rabies+JE-Conventional** | | **Rabies-Conventional** | | |
| --- | --- | --- | --- | --- | --- | --- | --- | --- |
|  | **N** | **% or value (95% CI)** |  | **N** | **% or value (95% CI)** |  | **N** | **% or value (95% CI)** |
| **Overall (Year 3–Year 10)** |  |  |  |  |  |  |  |  |
| RVNA (≥0.5 IU/mL) 7 days post-booster | 46 | 89.1 (76.4–96.4) |  | 43 | 95.3 (84.2–99.4) |  | 52 | 98.1 (89.7–100) |
| RVNA (≥0.5 IU/mL) 6–9 months post-booster | 45 | 97.8 (88.2–99.9) |  | 44 | 97.7 (88.0–99.9) |  | 52 | 96.2 (86.8–99.5) |
| GMC (IU/mL) 7 days post-booster | 46 | 4.2 (2.8–6.2) |  | 43 | 4.2 (2.8–6.4) |  | 52 | 4.4 (3.0–6.4) |
| GMC (IU/mL) 6–9 months post-booster | 45 | 12.4 (7.4–20.8) |  | 44 | 11.9 (7.0–20.1) |  | 52 | 10.1 (6.2–16.4) |
| GMR 7 days post-booster/pre-booster | 46 | 23.4 (16.2–33.8) |  | 43 | 23.6 (16.1–34.4) |  | 52 | 19.3 (13.7–27.3) |
| GMR 6–9 months post-booster/pre-booster | 45 | 69.9 (44.4–109.8) |  | 44 | 65.2 (41.3–103.0) |  | 52 | 43.8 (28.8–66.7) |
| **Year 3** |  |  |  |  |  |  |  |  |
| RVNA (≥0.5 IU/mL) 7 days post-booster | 30 | 83 (65–94) |  | 23 | 96 (78–100) |  | 25 | 96 (80–100) |
| RVNA (≥0.5 IU/mL) 6–9 months post-booster | 30 | 97 (83–100) |  | 23 | 100 (85–100) |  | 24 | 96 (79–100) |
| GMC (IU/mL) 7 days post-booster | 30 | 3.0 (1.8–5.1) |  | 23 | 3.7 (2.0–6.8) |  | 25 | 3.4 (1.9–6.1) |
| GMC (IU/mL) 6–9 months post-booster | 30 | 7.0 (3.8–12.7) |  | 23 | 6.9 (3.5–13.6) |  | 24 | 6.2 (3.2–12.1) |
| GMR 7 days post-booster/pre-booster | 30 | 20.7 (13.0–32.9) |  | 23 | 27.4 (16.1–46.5) |  | 25 | 17.7 (10.6–29.3) |
| GMR 6–9 months post-booster/pre-booster | 30 | 48.0 (29.0–79.3) |  | 23 | 51.0 (28.7–90.6) |  | 24 | 31.1 (17.7–54.7) |
| **Year 4** |  |  |  |  |  |  |  |  |
| RVNA (≥0.5 IU/mL) 7 days post-booster | 10 | 100 (69–100) |  | 8 | 88 (47–100) |  | 14 | 100 (77–100) |
| RVNA (≥0.5 IU/mL) 6–9 months post-booster | 10 | 100 (69–100) |  | 8 | 100 (63–100) |  | 14 | 100 (77–100) |
| GMC (IU/mL) 7 days post-booster | 10 | 6.2 (3.0–12.9) |  | 8 | 4.3 (1.9–9.7) |  | 14 | 4.9 (2.7–9.1) |
| GMC (IU/mL) 6–9 months post-booster | 10 | 35.7 (12.5–102.2) |  | 8 | 22.3 (6.9–72.3) |  | 14 | 17.7 (7.3–43.1) |
| GMR 7 days post-booster/pre-booster | 10 | 24.9 (11.6–53.4) |  | 8 | 23.0 (9.8–53.9) |  | 14 | 20.0 (10.5–38.1) |
| GMR 6–9 months post-booster/pre-booster | 10 | 143.3 (54.1–379.7) |  | 8 | 119.8 (40.3–356.0) |  | 14 | 72.1 (31.6–164.3) |
| **Year 5** |  |  |  |  |  |  |  |  |
| RVNA (≥0.5 IU/mL) 7 days post-booster | 1 | 100 (3–100) |  | 8 | 100 (63–100) |  | 3 | 100 (29–100) |
| RVNA (≥0.5 IU/mL) 6–9 months post-booster | 1 | 100 (3–100) |  | 9 | 89 (52–100) |  | 3 | 100 (29–100) |
| GMC (IU/mL) 7 days post-booster | 1 | 14.0 (0.5–371.9) |  | 8 | 4.1 (1.3–13.1) |  | 3 | 3.6 (0.5–23.6) |
| GMC (IU/mL) 6–9 months post-booster | 1 | 8.6 (0.1–785.5) |  | 9 | 11.2 (2.5–50.3) |  | 3 | 11.3 (0.8–153.1) |
| GMR 7 days post-booster/pre-booster | 1 | 46.7 (1.8–1207.4) |  | 8 | 13.1 (4.2–41.5) |  | 3 | 11.2 (1.7–73.1) |
| GMR 6–9 months post-booster/pre-booster | 1 | 28.7 (0.4–1996.2) |  | 9 | 35.8 (8.7–147.5) |  | 3 | 35.6 (3.1–412.3) |
| **Year 6** |  |  |  |  |  |  |  |  |
| RVNA (≥0.5 IU/mL) 7 days post-booster | 3 | 100 (29–100) |  | 3 | 100 (29–100) |  | 8 | 100 (63–100) |
| RVNA (≥0.5 IU/mL) 6–9 months post-booster | 2 | 100 (16–100) |  | 3 | 100 (29–100) |  | 9 | 89 (52–100) |
| GMC (IU/mL) 7 days post-booster | 3 | 7.2 (1.0–53.2) |  | 3 | 13.3 (1.8–98.3) |  | 8 | 7.3 (2.1–24.7) |
| GMC (IU/mL) 6–9 months post-booster | 2 | 26.3 (2.5–277.6) |  | 3 | 146.0 (21.3–1000.2) |  | 9 | 11.7 (3.8–35.4) |
| GMR 7 days post-booster/pre-booster | 3 | 28.6 (4.0–202.7) |  | 3 | 46.2 (6.5–327.2) |  | 8 | 27.6 (8.3–91.5) |
| GMR 6–9 months post-booster/pre-booster | 2 | 93.0 (8.4–1032.2) |  | 3 | 506.2 (70.9–3613.2) |  | 9 | 43.6 (14.0–135.7) |
| **Year 7** |  |  |  |  |  |  |  |  |
| RVNA (≥0.5 IU/mL) 7 days post-booster | 1 | 100 (3–100) |  | 0 |  |  | 0 |  |
| RVNA (≥0.5 IU/mL) 6–9 months post-booster | 1 | 100 (3–100) |  | 0 |  |  | 0 |  |
| GMC (IU/mL) 7 days post-booster | 1 | 44.0 |  | 0 |  |  | 0 |  |
| GMC (IU/mL) 6–9 months post-booster | 1 | 1480.2 |  | 0 |  |  | 0 |  |
| GMR 7 days post-booster/pre-booster | 1 | 110.0 |  | 0 |  |  | 0 |  |
| GMR 6–9 months post-booster/pre-booster | 1 | 3700.5 |  | 0 |  |  | 0 |  |
| **Year 9** |  |  |  |  |  |  |  |  |
| RVNA (≥0.5 IU/mL) 7 days post-booster | 1 | 100 (3–100) |  | 1 | 100 (3–100) |  | 2 | 100 (16–100) |
| RVNA (≥0.5 IU/mL) 6–9 months post-booster | 1 | 100 (3–100) |  | 1 | 100 (3–100) |  | 2 | 100 (16–100) |
| GMC (IU/mL) 7 days post-booster | 1 | 6.0 (1.1–33.8) |  | 1 | 3.9 (0.7–22.0) |  | 2 | 7.3 (2.1–24.7) |
| GMC (IU/mL) 6–9 months post-booster | 1 | 22.0 (0.3–1501.0) |  | 1 | 20.0 (0.3–1364.5) |  | 2 | 31.6 (1.6–626.3) |
| GMR 7 days post-booster/pre-booster | 1 | 30.0 (0.3–2698.6) |  | 1 | 13.0 (0.1–1169.4) |  | 2 | 25.7 (1.1–618.7) |
| GMR 6–9 months post-booster/pre-booster | 1 | 110.0 (14.8–816.8) |  | 1 | 66.7 (9.0–495.0) |  | 2 | 111.8 (27.1–461.5) |

PCEC, purified chick embryo cell; Rabies+JE-Accelerated, participants who received rabies vaccine concomitantly with Japanese encephalitis vaccine according to the accelerated one-week schedule; Rabies+JE-Conventional, participants who received rabies vaccine concomitantly with Japanese encephalitis vaccine according to the conventional four-week schedule; Rabies-Conventional, participants who received rabies vaccine alone according to the conventional four-week schedule; N, total number of participants for each timepoint; 95% CI, 95% confidence interval; RVNA, rabies virus neutralizing antibody; IU, international units; GMC, geometric mean concentration; GMR, geometric mean ratio.

Note: No PCEC rabies vaccine booster dose was administered at year 8.
