# Supplementary material for: Long-term persistence and boostability of immune responses following different rabies pre-exposure prophylaxis priming schedules of a purified chick embryo cell rabies vaccine administered alone or concomitantly with a Japanese encephalitis vaccine
Source: PLoS Negl Trop Dis. 2025 May 27;19(5):e0013118. doi: 10.1371/journal.pntd.0013118 (PMC12136438; doi:10.1371/journal.pntd.0013118)
Supplement: S5 Table — (DOCX) [file pntd.0013118.s007.docx]

## S5 Table. Boostability of the PCEC rabies vaccine (per-protocol set 1)

| **Timepoint** | **Rabies+JE-Accelerated** | | | **Rabies+JE-Conventional** | | | **Rabies-Conventional** | | |
| --- | --- | --- | --- | --- | --- | --- | --- | --- | --- |
|  | **N** | **n** | **% or value (95% CI)** | **N** | **n** | **% or value (95% CI)** | **N** | **n** | **% or value (95% CI)** |
| **Overall** |  |  |  |  |  |  |  |  |  |
| RVNA (≥0.5 IU/mL) 7 days post-booster | 36 | 32 | 89 (74–97) | 34 | 34 | 100 (90–100) | 40 | 39 | 98 (87–100) |
| RVNA (≥0.5 IU/mL) 6–9 months post-booster | 35 | 34 | 97 (85–100) | 35 | 34 | 97 (85–100) | 41 | 39 | 95 (83–99) |
| GMC (IU/mL) 7 days post-booster | 36 |  | 4.0 (2.6–6.2) | 34 |  | 4.9 (3.1–7.7) | 40 |  | 4.4 (2.9–6.7) |
| GMC (IU/mL) 6–9 months post-booster | 35 |  | 12.5 (6.8–22.9) | 35 |  | 12.2 (6.7–22.3) | 41 |  | 11.7 (6.7–20.4) |
| GMR 7 days post-booster/pre-booster | 36 |  | 23.1 (15.5–34.4) | 34 |  | 26.4 (17.5–39.8) | 40 |  | 18.0 (12.3–26.2) |
| GMR 6–9 months post-booster/pre-booster | 35 |  | 72.6 (42.7–123.4) | 35 |  | 65.4 (38.5–111.1) | 41 |  | 47.2 (28.9–77.1) |
| **Year 3** |  |  |  |  |  |  |  |  |  |
| RVNA (≥0.5 IU/mL) 7 days post-booster | 24 | 20 | 83 (63–95) | 18 | 18 | 100 (81–100) | 16 | 15 | 94 (70–100) |
| RVNA (≥0.5 IU/mL) 6–9 months post-booster | 24 | 23 | 96 (79–100) | 18 | 18 | 100 (81–100) | 16 | 15 | 94 (70–100) |
| GMC (IU/mL) 7 days post-booster | 24 |  | 2.7 (1.5–4.8) | 18 |  | 4.6 (2.4–8.9) | 16 |  | 3.1 (1.5–6.2) |
| GMC (IU/mL) 6–9 months post-booster | 24 |  | 7.8 (3.8–16.0) | 18 |  | 7.3 (3.2–16.8) | 16 |  | 8.2 (3.4–19.6) |
| GMR 7 days post-booster/pre-booster | 24 |  | 18.5 (11.4–30.0) | 18 |  | 35.0 (20.0–61.4) | 16 |  | 13.9 (7.6–25.1) |
| GMR 6–9 months post-booster/pre-booster | 24 |  | 53.2 (29.1–97.0) | 18 |  | 56.0 (28.0–112.2) | 16 |  | 36.9 (17.7–77.0) |
| **Year 4** |  |  |  |  |  |  |  |  |  |
| RVNA (≥0.5 IU/mL) 7 days post-booster | 6 | 6 | 100 (54–100) | 6 | 6 | 100 (54–100) | 12 | 12 | 100 (74–100) |
| RVNA (≥0.5 IU/mL) 6–9 months post-booster | 6 | 6 | 100 (54–100) | 6 | 6 | 100 (54–100) | 12 | 12 | 100 (74–100) |
| GMC (IU/mL) 7 days post-booster | 6 |  | 7.1 (3.0–16.6) | 6 |  | 6.6 (2.8–15.6) | 12 |  | 5.0 (2.7–9.1) |
| GMC (IU/mL) 6–9 months post-booster | 6 |  | 28.2 (7.0–113.7) | 6 |  | 28.0 (6.9–113.0) | 12 |  | 9.2 (7.2–51.4) |
| GMR 7 days post-booster/pre-booster | 6 |  | 33.1 (13.3–82.2) | 6 |  | 28.9 (11.6–72.0) | 12 |  | 20.1 (10.6–38.3) |
| GMR 6–9 months post-booster/pre-booster | 6 |  | 131.7 (35.4–489.6) | 6 |  | 122.4 (32.9–455.0) | 12 |  | 77.9 (30.8–197.3) |
| **Year 5** |  |  |  |  |  |  |  |  |  |
| RVNA (≥0.5 IU/mL) 7 days post-booster | 1 | 1 | 100 (3–100) | 6 | 6 | 100 (54–100) | 3 | 3 | 100 (29–100) |
| RVNA (≥0.5 IU/mL) 6–9 months post-booster | 1 | 1 | 100 (3–100) | 7 | 6 | 86 (42–100) | 3 | 3 | 100 (29–100) |
| GMC (IU/mL) 7 days post-booster | 1 |  | 14.0 (0.9–217.9) | 6 |  | 2.7 (0.9–8.2) | 3 |  | 3.6 (0.7–17.3) |
| GMC (IU/mL) 6–9 months post-booster | 1 |  | 8.6 (0.1–1038.7) | 7 |  | 7.1 (1.2–43.6) | 3 |  | 11.3 (0.7–179.9) |
| GMR 7 days post-booster/pre-booster | 1 |  | 46.7 (3.3–667.1) | 6 |  | 8.8 (3.0–26.1) | 3 |  | 11.2 (2.4–51.9) |
| GMR 6–9 months post-booster/pre-booster | 1 |  | 28.7 (0.3–2580.7) | 7 |  | 23.5 (4.3–129.0) | 3 |  | 35.6 (2.6–478.2) |
| **Year 6** |  |  |  |  |  |  |  |  |  |
| RVNA (≥0.5 IU/mL) 7 days post-booster | 3 | 3 | 100 (29–100) | 3 | 3 | 100 (29–100) | 7 | 7 | 100 (59–100) |
| RVNA (≥0.5 IU/mL) 6–9 months post-booster | 2 | 2 | 100 (16–100) | 3 | 3 | 100 (29–100) | 8 | 7 | 88 (47–100) |
| GMC (IU/mL) 7 days post-booster | 3 |  | 7.2 (0.9–58.7) | 3 |  | 13.3 (1.6–108.4) | 7 |  | 8.1 (2.1–31.9) |
| GMC (IU/mL) 6–9 months post-booster | 2 |  | 26.3 (2.7–254.0) | 3 |  | 146.0 (22.9–930.1) | 8 |  | 9.1 (2.9–28.2) |
| GMR 7 days post-booster/pre-booster | 3 |  | 28.6 (3.6–226.4) | 3 |  | 46.2 (5.8–365.3) | 7 |  | 29.6 (7.6–114.5) |
| GMR 6–9 months post-booster/pre-booster | 2 |  | 93.0 (9.8–882.9) | 3 |  | 506.2 (80.6–3180.5) | 8 |  | 32.8 (10.6–100.9) |
| **Year 7** |  |  |  |  |  |  |  |  |  |
| RVNA (≥0.5 IU/mL) 7 days post-booster | 1 | 1 | 100 (3–100) | 0 | 0 |  | 0 | 0 |  |
| RVNA (≥0.5 IU/mL) 6–9 months post-booster | 1 | 1 | 100 (3–100) | 0 | 0 |  | 0 | 0 |  |
| GMC (IU/mL) 7 days post-booster | 1 |  | 44.0 | 0 |  |  | 0 |  |  |
| GMC (IU/mL) 6–9 months post-booster | 1 |  | 1480.2 | 0 |  |  | 0 |  |  |
| GMR 7 days post-booster/pre-booster | 1 |  | 110.0 | 0 |  |  | 0 |  |  |
| GMR 6–9 months post-booster/pre-booster | 1 |  | 3700.5 | 0 |  |  | 0 |  |  |
| **Year 9** |  |  |  |  |  |  |  |  |  |
| RVNA (≥0.5 IU/mL) 7 days post-booster | 1 | 1 | 100 (3–100) | 1 | 1 | 100 (3–100) | 2 | 2 | 100 (16–100) |
| RVNA (≥0.5 IU/mL) 6–9 months post-booster | 1 | 1 | 100 (3–100) | 1 | 1 | 100 (3–100) | 2 | 2 | 100 (16–100) |
| GMC (IU/mL) 7 days post-booster | 1 |  | 6.0 (1.1–33.8) | 1 |  | 3.9 (0.7–22.0) | 2 |  | 7.3 (2.1–24.7) |
| GMC (IU/mL) 6–9 months post-booster | 1 |  | 22.0 (0.3–1501.0) | 1 |  | 20.0 (0.3–1364.5) | 2 |  | 31.6 (1.6–626.3) |
| GMR 7 days post-booster/pre-booster | 1 |  | 30.0 (0.3–2698.6) | 1 |  | 13.0 (0.1–1169.4) | 2 |  | 25.7 (1.1–618.7) |
| GMR 6–9 months post-booster/pre-booster | 1 |  | 110.0 (14.8–816.8) | 1 |  | 66.7 (9.0–495.0) | 2 |  | 111.8 (27.1–461.5) |

PCEC, purified chick embryo cell; Rabies+JE-Accelerated, participants who received rabies vaccine concomitantly with Japanese encephalitis vaccine according to the accelerated one-week schedule; Rabies+JE-Conventional, participants who received rabies vaccine concomitantly with Japanese encephalitis vaccine according to the conventional four-week schedule; Rabies-Conventional, participants who received rabies vaccine alone according to the conventional four-week schedule; N, total number of participants for each timepoint; n (%), number (percentage) of participants in a given category; 95% CI, 95% confidence interval; RVNA, rabies virus neutralizing antibody; IU, international units; GMC, geometric mean concentration; GMR, geometric mean ratio.

Note: No PCEC rabies vaccine booster dose was administered at year 8.
