# Supplementary material for: Long-term persistence and boostability of immune responses following different rabies pre-exposure prophylaxis priming schedules of a purified chick embryo cell rabies vaccine administered alone or concomitantly with a Japanese encephalitis vaccine
Source: PLoS Negl Trop Dis. 2025 May 27;19(5):e0013118. doi: 10.1371/journal.pntd.0013118 (PMC12136438; doi:10.1371/journal.pntd.0013118)
Supplement: S6 Table — (DOCX) [file pntd.0013118.s008.docx]

## S6 Table. Antibody persistence after booster dose using repeated measures – yearly GMCs (full analysis set 1)

| **Years after booster dose** | **GMC (IU/mL; 95% CI)** | | | |
| --- | --- | --- | --- | --- |
|  | **Rabies+JE-Accelerated** | **Rabies+JE-Conventional** | **Rabies-Conventional** | **Pooled regimens** |
| 1 year | 9.53 (5.44–16.68) | 13.19 (7.15–24.36) | 9.36 (5.43–16.14) | 10.56 (7.41–15.04) |
| 2 years | 7.30 (4.16–12.8) | 7.05 (3.81–13.05) | 6.26 (3.62–10.81) | 6.86 (4.81–9.78) |
| 3 years | 5.41 (3.08–9.50) | 5.47 (2.96–10.13) | 4.88 (2.82–8.43) | 5.25 (3.68–7.49) |
| 4 years | 4.98 (2.83–8.76) | 4.89 (2.64–9.07) | 4.28 (2.48–7.4) | 4.71 (3.30–6.72) |
| 5 years | 4.81 (2.73–8.49) | 4.49 (2.42–8.34) | 4.32 (2.49–7.50) | 4.54 (3.17–6.49) |
| 6 years | 4.33 (2.44–7.67) | 3.97 (2.13–7.41) | 3.48 (2.00–6.05) | 3.91 (2.73–5.60) |
| 7 years | 4.30 (2.41–7.67) | 3.86 (2.05–7.26) | 4.04 (2.28–7.17) | 4.06 (2.82–5.86) |

GMC, geometric mean concentration; IU, international units; 95% CI, 95% confidence interval; Rabies+JE-Accelerated, participants who received rabies vaccine concomitantly with Japanese encephalitis vaccine according to the accelerated one-week schedule; Rabies+JE-Conventional, participants who received rabies vaccine concomitantly with Japanese encephalitis vaccine according to the conventional four-week schedule; Rabies-Conventional, participants who received rabies vaccine alone according to the conventional four-week schedule.
